# Investigating the psychometric properties of the Persian version of the attitude scale for cancer screening

**DOI:** 10.1186/s12889-023-16981-1

**Published:** 2023-10-23

**Authors:** Naeimeh Sarkhani, Reza Negarandeh, Mohammad Ehsan Heshmatian

**Affiliations:** 1 Nursing and Midwifery Care Research Center, School of Nursing and Midwifery, Tehran University of Medical Sciences, Tehran, Iran; 2 Research Center for Cognitive & Behavioral Sciences in Police, Directorate of Health, Rescue & Treatment, Police Headquarter, Tehran, Iran

**Keywords:** Attitude, Cancer screening, Psychometrics, Scale

## Abstract

**Background:**

Individual attitude is an essential component in facilitating people’s participation in adopting cancer screening behaviors. People’s attitudes toward cancer screening should be evaluated with a valid and reliable scale. Therefore, this study investigated the psychometric properties of the Persian version of the attitude scale for cancer screening.

**Method:**

In this psychometric study, the Farsi version of the attitude scale for cancer screening was prepared from English to Farsi using the Backward-Forward method. Then content, face, and construct validity, plus reliability, was evaluated by Internal Consistency and Stability methods. Construct validity was investigated using Exploratory Factor Analysis with a sample of 246 adults referring to health centers in the south of Tehran. Data analysis was done using SPSS V24 software.

**Results:**

All items received an acceptable Content Validity Ratio. The content Validity Index was confirmed for all items with a value greater than 0.79. In the qualitative review of content and face validity, all items were confirmed. Unlike the original version of the scale, which was a single factor, the results of Exploratory Factor Analysis indicated the existence of 2 factors explaining 63.84% of the total variance of the scale. Reliability based on Cronbach’s alpha coefficient for the whole scale was 0.88, and Intraclass Correlation Coefficient for the entire scale was calculated for scale reliability using the single-rating, absolute-agreement, 2-way mixed-effects method as 0.84.

**Conclusion:**

This study confirmed the validity and reliability of the Persian version of the attitude scale for cancer screening with two factors (Willing Attitude and Unwilling Attitude). Therefore, it can be a suitable and valid scale to evaluate the attitude of the community for cancer screening.

## Introduction

Cancer is a leading global health issue and the second leading cause of death, with over 19.3 million cases in 2020 and an expected rise to 30.2 million by 2040 [1, 2]. Iran recorded over 79 thousand cancer-related deaths in 2020, with breast, stomach, lung, prostate, and colorectal cancers making up about 45% of the cancer burden [2, 3].

Cancer prevention is possible for 30–50% of cases by avoiding risk factors and implementing evidence-based prevention strategies [4]. This makes cancer screening crucial, especially in low-income countries where comprehensive treatment is less than 15% available [5, 6]. The World Health Organization (WHO) recommends population-based screening programs for early detection of breast, cervical, and colorectal cancer [7]. The “National Cancer Screening Program” in Iran consists of screening for three types of cancer, including breast cancer, cervix cancer, and colorectal cancer. Breast and cervical cancer screening is performed in women in the age groups of 69 − 30 and 59 − 30, respectively, and colon cancer screening is performed in all people aged 50–69 [8]. All types of health services, such as tests and clinical examinations, which are performed before the appearance of signs and symptoms, mainly in the population at risk, are considered cancer screening [9]. Cancer screening aims to detect cancer or precancerous lesions before symptoms appear or before the cancer stage progresses and to start cancer treatment as soon as possible. People’s participation in cancer screening helps increase treatment effectiveness and reduce incidence and mortality [10].

Despite the availability of cancer screening methods, people’s acceptance of these facilities is low. According to the results of the studies, there are many obstacles related to screening and adopting cancer screening behaviors, among which individual attitudes can be mentioned [11, 12]. Fishbein and Ajzen (1975) define attitude as “a disposition to respond favorably or unfavorably towards some psychological object.“ Also, attitude means feelings, opinions, and relatively hidden behavioral contexts that are driven in the context of persons, groups, thoughts, or things [12, 13]. In a study conducted with 335 people in Tehran, the average attitude score in people who had screening was lower than in people who were not screened [14]. Also, a study conducted by Calpbinici and Öztoprak showed that the attitude for cancer screening is related to factors such as spiritual growth, responsibility for health, and interpersonal relationships [15]. Therefore, attitude is considered a socio-psychological factor in cancer prevention and screening behaviors. Thus, attitudes must be reliably measured to predict people’s behavior to participate in screening. By accurately measuring attitudes, effective interventions can be planned for people with positive or negative attitudes, and the cancer screening rate can be increased [12, 16].

Researchers need to measure public attitudes toward cancer screening with a valid and standardized scale [16]. In the review of the literature, it is observed that there are studies with scales in the field of attitude for cancer screening, but these scales measure the attitude for screening of one type of cancer [17, 18]. The only scale that measures the general attitude for cancer screening is the Attitude Scale for Cancer Screening (ASCS-15). The preliminary version of this scale with 24 items was validated by Öztürk and his colleagues in 2020 in Turkey; the developers then decided to remove 9 items based on the results of the confirmatory factor analysis (EFA). Finally, they proposed a valid and reliable 15-item scale (ASCS-15). It has been introduced as a self-assessment scale to evaluate the attitude for cancer screening in adults. This scale has good validity and reliability; the number of items is small, simple, and understandable. This scale measured the general attitude for cancer screening, and its psychometric properties were investigated in the Turkish adult population [9]. For health workers and Persian-speaking people to access the scale of attitudes for cancer screening (ASCS-15), it is necessary to translate this scale into Farsi and examine the psychometric features of the translated version. Therefore, the present study investigated the psychometric properties of the Persian version of the Attitude Scale for Cancer Screening (ASCS-P15) in Iranian adults.

## Method

This methodological study was conducted between April and July 2023 in Iran. First, the scale was translated into Persian, and then content, face, and construct validity were investigated. Reliability was also evaluated in terms of Internal Consistency and Stability. More details of each step are given below.

### Study population and sampling and sample size

The study population was all adults between 30 and 70 years living in the south of Tehran, and the study environment was comprehensive health service centers in the south of Tehran. Purposeful sampling was used for content and face validity and reliability. In contrast, multi-stage sampling was used for selecting samples for assessing construct validity. Since the comprehensive health centers in the south of Tehran are located in five regions, 10, 11, 16, 17, and 19, one center was randomly selected from each area, and the researcher went to these centers for sampling. After that, eligible samples were continuously selected from these centers. The inclusion criteria included people aged 30–70, not suffering from severe physical, mental, or learning disorders, and willing to participate in the study. There are two general recommendations regarding the minimum sample size required to perform factorial analysis; the first recommendation is based on the importance of an absolute number of cases (N), and the second recommendation expresses the importance of subject-to-variable ratio (p). In this regard, Guilford suggests that N should be at least 200 [19]. Also, MacCallum et al. recommended that the subject-to-variable ratio should be at least 5 [20]. Therefore, considering the non-response rate of 15%, 280 questionnaires were distributed, of which 246 people completed.

### ASCS-15

The ASCS-15, which was adapted from the ASCS-24, was developed in 2020 by Öztürk et al. in Turkey. It was specifically designed for people 30–70 years old. The developer of the scale assessed all the psychometric procedures associated with it. This single-factor scale has 15 items. The questions are based on a 5-point Likert scale (5: completely agree, 4: somewhat agree, 3: neither agree nor disagree, 2: somewhat disagree, 1: completely disagree). The range is from 15 to 75, with scores closer to 15 indicating a negative attitude for cancer screening, while scores closer to 75 indicate a positive attitude. Six items (10-15) should be reverse-coded when calculating scores. There is no specific cutoff point for it. ASCS-15 is a self-administered scale whose original language is Turkish, translated from Turkish to English [9].

### Translation of the ASCS-15 scale

After correspondence with Dr. Öztürk and get permission from him the scale was translated using the standard Backward-Forward method. Thus, the scale was first translated independently and simultaneously from English to Persian by two translators fluent in Farsi and English. In the second stage, the translations were placed next to each other and prepared as a single translation. In this way, translations were compared, and contradictions were identified, and corrections were applied based on the opinions of a group of experts (including four community health experts, one public health expert, two scale psychometric experts, and one nursing professor). In the third stage, the prepared Persian version was given to two translators who are fluent in Persian and English (independent of the translators of the first stage) to translate it from Persian to English, and thus the scale was translated from Persian to English. In the fourth stage, two translations were placed next to each other, and the contradictions were resolved by experts, and a single translation was prepared. In the last step, before finalizing the scale, the translated version was emailed to the scale designer to check and confirm the compatibility of the sent version with the original version. Then, the pre-final version was evaluated in the content and face validity process.

### Content validity

The validity of the content was evaluated using two qualitative and quantitative methods using a group of experts. In the content validity using a qualitative method, experts’ opinions were obtained about the appropriate position of the items, use of appropriate words, compliance with grammar, and proper scoring of the items and necessary guidelines, and their opinions were the basis of the necessary changes. Quantitative content validity was evaluated based on Lawshe’s Content Validity Ratio (CVR) and Waltz & Bausell’s Content Validity Index (CVI). To calculate CVR, the experts were asked to classify each of the questions based on the three-part Likert spectrum of “the item is necessary”, “the item is useful but not necessary” and “the item is not necessary”. Then, using the formula, CVR was calculated. In this formula, N and NE are equal to the total number of experts and the number of experts who rate the desired item as essential, respectively. In the CVI review, the same experts were asked to determine the degree of relevance of each item with a four-part spectrum: 1- not relevant, 2- somewhat relevant, 3- completely relevant, and 4- very relevant. The number of experts who chose options 3 and 4 was divided by the total number of experts. Items that obtain a value of 0.79 or more are suitable [21].

### Face validity

Face validity was evaluated from the point of view of the target group (8 adults referring to health centers) and the group of experts in content validity using a qualitative approach. The target group was asked to rate and comment on the appropriateness, difficulty, relevance, and ambiguity of the ASCS-P15 items. Also, the group of experts examined the expressions regarding the clarity of using simple and understandable words and the use of common language (avoiding technical and specialized words). The experts were asked to explain their opinions and suggestions before each item.

### Construct validity

Exploratory Factor Analysis (EFA) was used to check the construct validity of the scale. Using EFA, items of the scale that show the highest correlation with each other in each factor can be placed as the items used in explaining each factor of the scale. A central question in factor analysis is determining how many factors should be extracted and retained to explain as much of the data as possible. The number of factors to be retained with eigenvalues greater than or equal to 1 was indicated by a scree plot. The Kaiser-Meyer-Olkin sampling index (KMO) was performed to ensure the sample’s adequacy. Also, Bartlett’s test of sphericity was used to determine whether there is enough correlation between the scale items to integrate them and whether the obtained correlation matrix has a significant difference from zero [22].

### Reliability

Internal Consistency of the ASCS-P15 scale was checked with the help of Cronbach’s alpha coefficient in a sample of 246 people from the target group. An alpha coefficient of 0.70 has often been regarded as an acceptable threshold for reliability; however, 0.80 and 0.95 are preferred for the psychometric quality of scales [23].

To evaluate the stability of the test-retest method with a time interval of two weeks, a sample of 30 people from the target group was checked, and the scores obtained from these two stages were evaluated using the Intraclass Correlation Coefficient (ICC) test, single-rating, absolute model. -agreement, 2-way mixed-effects were calculated. The ICC values less than 0.5 indicate poor reliability, values between 0.5 and 0.75 indicate moderate reliability, values between 0.75 and 0.9 indicate good reliability and values greater than 0.90 indicate excellent reliability [24].

### Data collection scales

Data were collected and recorded through the Demographic Characteristics Scale and ASCS-P15. The demographic characteristics scale included six questions about age, gender, education level, marital status, employment status, and income.

### Ethical considerations

This study was evaluated and approved by the Research Ethics Committee of Tehran University of Medical Sciences (ethical code: IR.TUMS.MEDICINE.REC.1400.1298). After receiving the code of ethics, necessary permissions were obtained from the study environment officials. Permission to use the original scale was also obtained from the author. The target group was informed of their freedom to withdraw from the study, the confidentiality of their data, and the study’s objectives. Written informed consent was obtained from them.

### Data analysis

Mean (standard deviation) was used to describe quantitative variables, and frequency report (percentage) was used to describe qualitative variables. Data analysis was done in SPSS V24 software. Missing data for each item were replaced by the mean of responses to that item.

## Results

### Demographic information

The average age of the participants was 41.65 ± 10.52 years, and most of the participants were male (61%). Other information is provided in Table 1.

**Table 1:** Demographic characteristics of the participants

Characteristics			frequency		
			N		(%)
**Gender**	Female	96		39	
	Male	150		61	
**Level of Education**	Illiterate	7		2.8	
	Elementary	14		5.7	
	Diploma	88		35.8	
	University	136		55.3	
**Marital Status**	Single	47		19.1	
	Married	172		69.9	
	Divorced	22		8.9	
	Widow	5		2	
**Employment Status**	Employed	175		71.1	
	Unemployed	13		5.3	
	Housewife	43		17.5	
	Retired	14		5.7	
**Monthly Income**	Adequate	54		22	
	Inadequate	87		35.4	
	Reasonably adequate	101		41.1	
**Age, Mean (SD)**			41.65 (10.52)		

### Content validity and face validity

In checking the validity of the content through the qualitative method, based on the opinions obtained from the experts, the scale was revised, and the necessary corrections were applied to each item. For example, the word “want” was changed to the word “willing” and the phrase “soon” to “as soon as possible”. Also, item 12, with the phrase “I think cancer screening methods are embarrassing,“ was modified to “I think some cancer screening methods cause me embarrassment.“ In quantitative content validity research, according to Lawshe, when the number of experts is eight, the minimum acceptable CVR is 0.75. Accordingly, all items received an acceptable CVR value above 0.75. The CVI value of each item was more than 0.79. Also, the findings of qualitative face validity showed that the level of difficulty, appropriateness and ambiguity of the scale was approved by the experts and the target group.

### Construct validity

**Fig. 1:**
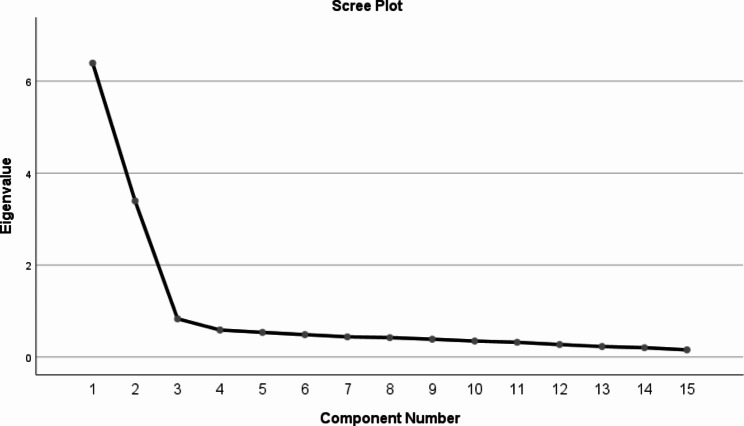
Scree plot for differentiated factors through exploratory factor analysis of ASCS-P15

The sampling adequacy index was calculated (KMO = 0.907 and Bartlett’s test = 2284.835, P < 0.001). In the scree plot (Fig. 1), factors with eigenvalues greater than one were evaluated, and two factors were extracted. Factor 1 includes nine items (items 1–9) which are named “Willing Attitude” and factor 2 includes six items (items 10–15) which are named “Unwilling Attitude”. These two factors explained a total of 63.84% of the total variance of ASCS-P15. All items had a factor loading above 0.6, ranging from 0.660 to 0.873, which means that all items have a common variance (Table 2). Also, Table 3 presents the maximum and minimum of participants’ responses to the scale’s items and their respective median, mean and standard deviation.

**Table 2:** Results of exploratory factor analysis of ASCS-P15 (n = 246)

Factor name	Items	Item content	% of variance	Factor Loading
**Willing Attitude**	1	I want to undergo cancer screening at regular intervals.	63.84	0.830
	2	I want to undergo cancer screening soon.		0.842
	3	I want to receive information about cancer screening tests.		0.802
	4	If there is anything I wonder about cancer screening, I will research it to find out.		0.873
	5	When I undergo a cancer screening test, I will follow the results.		0.660
	6	I encourage people around me to undergo cancer screening.		0.805
	7	Giving information about cancer screenings on television, on the internet, and in the newspaper has a positive effect on my screening.		0.814
	8	Cancer screening recommendations by a health worker increase my likelihood of being screened.		0.864
	9	When I undergo a cancer screening, I think that I’m doing something good for myself.		0.820
**Unwilling Attitude**	10	I think it’s unnecessary to undergo cancer screening.		0.744
	11	I think that I’m not the right age for cancer screening.		0.744
	12	I think the procedures of cancer screening are embarrassing.		0.793
	13	I don’t trust the results of cancer screening tests.		0.779
	14	I don’t need to undergo cancer screening because I think that cancer won’t happen to me.		0.829
	15	I have more important things to do than cancer screening.		0.752

**Table 3:** Scores of ASCS-P15 in the participants (n = 246)

Items	Item content	Maximum	Minimum	Median	Mean (SD)
1	I want to undergo cancer screening at regular intervals.	5	1	5	4.12 (1.19)
2	I want to undergo cancer screening soon.	5	1	4	4.03 (1.15)
3	I want to receive information about cancer screening tests.	5	1	5	4.35 (1.08)
4	If there is anything I wonder about cancer screening, I will research it to find out.	5	1	5	4.32 (0.98)
5	When I undergo a cancer screening test, I will follow the results.	5	1	5	4.57 (0.83)
6	I encourage people around me to undergo cancer screening.	5	1	5	4.23 (1.10)
7	Giving information about cancer screenings on television, on the internet, and in the newspaper has a positive effect on my screening.	5	1	4	4.12 (1.14)
8	Cancer screening recommendations by a health worker increase my likelihood of being screened.	5	1	5	4.24 (1.03)
9	When I undergo a cancer screening, I think that I’m doing something good for myself.	5	1	5	4.36 (0.98)
10	I think it’s unnecessary to undergo cancer screening.	5	1	5	4.07 (1.31)
11	I think that I’m not the right age for cancer screening.	5	1	4	3.72 (1.45)
12	I think the procedures of cancer screening are embarrassing.	5	1	5	3.93 (1.39)
13	I don’t trust the results of cancer screening tests.	5	1	5	3.98 (1.40)
14	I don’t need to undergo cancer screening because I think that cancer won’t happen to me.	5	1	5	4.21 (1.31)
15	I have more important things to do than cancer screening.	5	1	5	3.83 (1.48)
Total Score	246	75	31	63	62.1(11)

### Reliability

For the ASCS-P15 scale, Cronbach’s alpha obtained was 0.88, and Cronbach’s alpha for factors 1 and 2 was calculated as 0.93 and 0.87, respectively (Table 4). In addition, the ICC value for the entire scale was 0.84, indicating good reliability (Table 5).

**Table 4:** Cronbach’s ɑ coefficient of the ASCS-P15 (n = 246)

Dimensions	Number of items	Cronbach’s alpha
Willing Attitude	9	0.93
Unwilling Attitude	6	0.87
ASCS-P15	15	0.88

**Table 5:** Results of ICC using single-rating, absolute-agreement, 2-way random-effects model (n = 30)

Dimensions	ICC	95% confidence interval Lower Bound Upper Bound	F test with true value 0 Value df1 df2 Sig
Willing Attitude	0.98	0.96 0.99	62.667 26 26 0.000
Unwilling Attitude	0.72	0.41 0.87	3.861 27 27 0.000
ASCS-P15	0.84	0.65 0.93	6.833 25 25 0.000

## Discussion

The present study investigated the psychometric properties of ASCS-P15 and presented a valid scale for use in studies related to the attitude for cancer screening in the Iranian adult population. Overall, the results showed that the ASCS-P15 is a suitable and valid scale that can be used to assess the attitude for cancer screening among Farsi-speaking people.

ASCS-15 has been translated and used for the first time in Iran. According to our knowledge, the 24-item version of this scale (ASCS-24) has been used in several other studies in Turkey, but none of these studies have reported its validity [25–27]. Next, we compare ASCS-P15 and ASCS-15. In this study, the validity of the content was checked by both quantitative and qualitative methods, and all the items were retained. Also, the ASCS-P15 scale had good content validity based on the CVI and CVR values. While in ASCS-15, content validity is only qualitatively reported [9]. The face validity results in this study showed the desirability of the items as in ASCS-15.

The various ethnic groups may have different factor structures, so evaluating whether the scale is valid and reliable for the target population is important. Considering that it would be more appropriate to conduct an EFA first to introduce possible cultural differences in the adapting process [28] Therefore, the present study used EFA to check the construct validity. The findings of our study revealed a two-factor structure for the ASCS-P15 scale, whereas the ASCS-15 identified one factor [9]. The first factor identified in this study was Willing Attitude with nine items. These items show people’s desire to participate in cancer screening and are related to the desire to obtain information, perform, follow up, and continue performing the screening behavior. Another factor that was identified in the present study was Unwilling Attitude with 6 items. These items show people’s reluctance to participate in cancer screening and are related to the lack of awareness and unnecessary behavior of cancer screening.

Some studies have used the word Willing next to the word Attitude [29–31]. According to Kressin et al., the type of attitude for cancer screening has often been associated with the desire to do it [29]. Enthusiasm, positive attitude, and willingness can greatly contribute to cancer screening willingness and play an important role in health and cancer prevention. “Attitude” is a central concept in theories such as the Theory of Reasoned Action (TRA) and Theory of Planned Behavior (TPB) to predict the intention to perform a specific behavior. TRA has four main components: Belief, Attitude, Subjective Norms, and Intention, and the perceived behavior control component was added to TRA to make the TPB theory [13]. In both theories, attitude means how desirable, pleasant, useful, or enjoyable the desired behavior is for the person, which depends on the individual’s judgment about the effects and consequences of the behavior [32]. Attitude is so important that another thing that Fishbein and Ajzen discussed in TRA was the inclusion of other people’s attitudes in predicting behavioral intention [13]. Therefore, it can be said that by measuring Attitude through this scale, the probability of performing cancer screening behaviors in people can be predicted. Therefore, it is suggested that in future studies, this scale should be developed using TPB and TRA theories, and social norms and behavior control should be measured as scale dimensions.

The reliability of the ASCS-P15 scale was calculated using Cronbach’s alpha coefficient and ICC, and the results show the good reliability of the scale. Reliability in ASCS-15 is limited to the calculation of Cronbach’s alpha, split-half, and Guttman coefficients, which are reported as 0.97, 0.94, and 0.94, respectively [9]. Similarly, in other studies that used the ASCS-24, they reported reliability only by calculating Cronbach’s alpha coefficient [25–27]. Since stability is a key feature in psychometric studies and a strong scale should show good test-retest reliability, this study used the test-retest method to evaluate stability [23].

Filling the gap of the limitation of the existence of a valid and reliable scale, which can be easily used to measure the attitude for cancer screening in the Farsi-speaking community, and the acceptable sample size are the strengths of this study. Despite its strengths, the present study had limitations. This study was conducted only in comprehensive health centers in the south of Tehran. Therefore, its generalization should be done with caution. Some participants did not complete the scale themselves due to illiteracy, which may cause bias in the results. To overcome some of these limitations, we recommend that future studies be conducted in different regions of the country and that the scales be completed by the participants themselves. Since there was no valid similar scale in Farsi and other languages, it was impossible to compare ASCS-P15 with other scales and evaluate Criterion Validity.

### Implications

The ASCS-P15 is a valuable tool for assessing the attitudes of adults toward cancer screening. It can be administered by healthcare providers such as nurses and physicians in various settings, such as health organizations and early cancer screening centers. The use of ASCS-P15 in extensive studies can provide significant benefits for cancer prevention and control. By applying ASCS-P15, healthcare providers can identify individuals who have negative attitudes toward cancer screening and implement appropriate interventions to enhance their positive attitudes, increase their screening behaviors, and ultimately reduce the risk of cancer morbidity and mortality.

## Conclusion

The present study evaluated the psychometric properties of ASCS-P15, a scale for measuring attitudes for cancer screening in adults aged 30 to 70. The results indicated that the scale had satisfactory content validity, face validity, construct validity, and reliability. The ASCS-P15 can be a useful instrument for researchers and healthcare providers who aim to assess and improve the attitudes of adults toward cancer screening.

## Data Availability

The data that support the findings of this study are available from the corresponding author, [**Reza Negarandeh**], upon reasonable request.
